# Effect of histone demethylase KDM5B on long-term cognitive impairment in neonatal rats induced by sevoflurane

**DOI:** 10.3389/fnmol.2024.1459358

**Published:** 2024-11-27

**Authors:** Yanhong Wang, Yun Chen, Mengxiao Zhang, Chengdong Yuan, Yu Zhang, Xingjian Liu, Yi Zhang, Xiaoli Liang

**Affiliations:** ^1^Department of Anesthesiology, Second Affiliated Hospital of Zunyi Medical University, Zunyi, China; ^2^Department of Anesthesiology, Xishui County People’s Hospital, Zunyi, China; ^3^Guizhou Key Laboratory of Anesthesia and Organ Protection, Zunyi Medical University, Zunyi, China; ^4^School of Anesthesiology, Zunyi Medical University, Zunyi, China

**Keywords:** sevoflurane, cognitive, histone methylation, hippocampus, neonatal

## Abstract

**Introduction:**

Whether repeated inhalation of sevoflurane during the neonatal period causes long-term learning and memory impairments in humans is unclear. Some recent investigations have indicated that general anesthesia drugs affect histone methylation modification and may further affect learning and memory ability. This study aimed to explore the role and mechanism of histone methylation in long-term cognitive dysfunction caused by repeated inhalation of sevoflurane during the neonatal period.

**Methods:**

Neonatal SD rats were assigned into three groups. Sevoflurane group and sevoflurane +AS8351 group were exposed to 2% sevoflurane for 4 h on postnatal day 7 (P7), day 14 (P7) and day 21 (P21), and the control group was inhaled the air oxygen mixture at the same time. From postnatal day 22 to 36, rats in the +AS8351 group were treated with AS8351 while those in the Sevoflurane group and control group were treated with normal saline. Half of the rats were carried out Y-maze, Morris water maze (MWM), western blot and transmission electron microscope at P37, and the remaining rats were fed to P97 for the same experiment.

**Results:**

Neonatal sevoflurane exposure affected histone demethylase expression in hippocampus, changed histone methylation levels, Down-regulated synapse-associated protein expression, impaired synaptic plasticity and long-term cognitive dysfunction and KDM5B inhibitors partially restored the negative reaction caused by sevoflurane exposure.

**Discussion:**

In conclusion, KDM5B inhibitor can save the long-term learning and memory impairment caused by sevoflurane exposure in neonatal period by inhibiting KDM5B activity.

## 1 Introduction

Sevoflurane is an anesthetic widely used in pediatric general anesthesia, whether it is safe to application to the developing brain has been widely concerned by medical experts and the public (Rappaport et al., 2011). A previous large retrospective study showed that children who repeatedly received general anesthesia had a higher incidence of learning disabilities and reasoning deficits (Zhao et al., 2020). Inhalation of sevoflurane during the neonatal period can cause a decline in rodents’ learning and memory ability (Goyagi, 2019; Zhang et al., 2022). Other studies have found that exposure to sevoflurane during neonatal primate life changes the transcriptional level of the gene locus of synaptic development and leads to neurodevelopmental defects (Chen B. et al., 2022). Whether sevoflurane exposure causes long-term cognitive impairment in the developing brain and its mechanism is still unclear, and new strategies are needed to explore it.

The amino tail of histones can reshape chromatin and regulate gene expression through various covalent modifications (Rothbart and Strahl, 2014). Among them, histone 3 lysine 4 trimethylated (H3K4me3) is mainly involved in nervous system development, learning, synaptic assembly, and synaptic plasticity (Park et al., 2020). Genetic studies have found that cognitive impairment in the nervous system and mental disorders during human development is closely related to the level of H3K4me3 (Collins et al., 2019), and H3K4me3 can affect learning, memory, and cognitive abilities by affecting synaptic plasticity of nerve cells (Maity et al., 2021; Jarome et al., 2021). Therefore, we speculate that H3K4me3 has an impact on learning, memory, and cognitive abilities during development by affecting synaptic plasticity in nerve cells.

Histone lysine methyltransferases (HKMTs) and histone lysine demethylases (HKDMs) jointly regulate the level of histone methylation (Al Ojaimi et al., 2022). HKDMs catalyze the methylation removal of lysine residues, in which lysine demethylase 5B (KDM5B) can specifically regulate H3K4me2/me3 levels, regulate gene transcription and cell differentiation (Li et al., 2020), and these effects may have a certain impact on neural function during development.

So far, people’s attention to histone methylation modification has mainly focused on differentiation, development, regeneration, and cancer (Shpargel et al., 2012; Morales Torres et al., 2013; Chakraborty et al., 2019; Revia et al., 2022), and there are few studies on its cognitive impairment during general anesthesia. Recent studies have shown that general anesthesia drugs can cause changes in histone methylation levels and may further negatively affect learning and memory ability (Wu et al., 2019; Holtkamp et al., 2019; Rump et al., 2022; Bhuiyan et al., 2022). However, whether sevoflurane exposure in neonatal rats has an effect on long-term learning and memory through histone methylation modification remains unclear.

Therefore, our study is based on the perspectives of behavior, synaptic structure, and molecular level comprehensively observed the effects of repeated sevoflurane inhalation on learning and memory, the protein expression levels of BDNF, PSD95, KDM5B and H3K4me3 in the hippocampus of rats, and the synaptic ultrastructure of hippocampus. Finally, observe whether treatment with histone demethylase inhibitors has an effect on the aforementioned changes. From the perspective of epigenetics, this study aims to explore the neural and molecular mechanisms underlying the long-term cognitive impairment caused by sevoflurane inhalation in neonatal rats, providing a theoretical basis for preventing the damage of inhaled anesthetics to the developing brain.

## 2 Materials and methods

### 2.1 Animals

In this experiment, healthy newborn Sprague Dawley (SPF-grade SD) rats were selected as the research objects. Pregnant rats were provided by the Animal Center of Zunyi Medical University, and animal license number was SYXK (Qian) 2001–0004. fter birth, the young mice were raised together with their mothers in the Animal Experimental Center of Zunyi Medical University. The animal room was set to the circadian rhythm of rats: 12 h of light (20:00–8:00) and 12 h of darkness (8,00–20:00), maintaining a room temperature of 23 ± 2°C and a relative humidity of 55 ± 5%. The animals were allowed to move, eat and drink water freely during the entire experiment and the bedding material was changed twice a week. All experimental procedures comply with the regulations of the Animal Ethics Review Committee of Zunyi Medical University.

### 2.2 Model establishment and grouping

For the statistical analyses of behavior test data with relatively large standard deviation, a total of 120 healthy neonatal SD rats were selected and divided into three groups: control group (CON group), sevoflurane group (SEV group), and sevoflurane + KDM5B inhibitor AS8351 group (SEV + AS8351 group), with 40 rats in each group. Except the CON group, rats in the other groups were inhaled 2% sevoflurane (with a carrier gas of 1 L/min of oxygen and 1 L/min of air, from 08:00 a.m. to 12: 00 a.m.) for 4 h on postnatal day 7 (Postnatal day 7, P7), day 14 (Postnatal day 14, P14), and day 21 (Postnatal day 21, P21). Continuously monitor the temperature inside the box and control it at about 27°C. During the treatment, the rats maintain natural breathing, adjusts their position to a lateral position, and extends their tongue out of the corner of the mouth to prevent them from causing airway obstruction. The nasal lip, distal extremities and tail of rats were closely observed for cyanosis, and the respiratory rate of rats was measured every half an hour. After the treatment was completed, the rats were placed back into the cage, and the standard of wakefulness after anesthesia is their movement, breathing, and reaction returned to normal. CON group rats inhaled the same carrier gas (1 L/min oxygen+1 L/min air) for 4 h at the same time. The rats that had completed the intervention were given daily intraperitoneal injection since P22: the SEV + AS8351 group was given KDM5B inhibitor AS8351 (NSC51355, MedChemexpress, USA) 2 mg/kg; the SEV group and the CON group were given Normal saline (NS) for 15 days. 20 rats in each group were randomly selected for behavioral, protein expression detection and Transmission electron microscope (TEM), and the remaining rats were kept in the animal house until day 97 according to the previous feeding conditions, and the same experimental procedures were performed.

### 2.3 Y maze experiment

**Figure 1:**
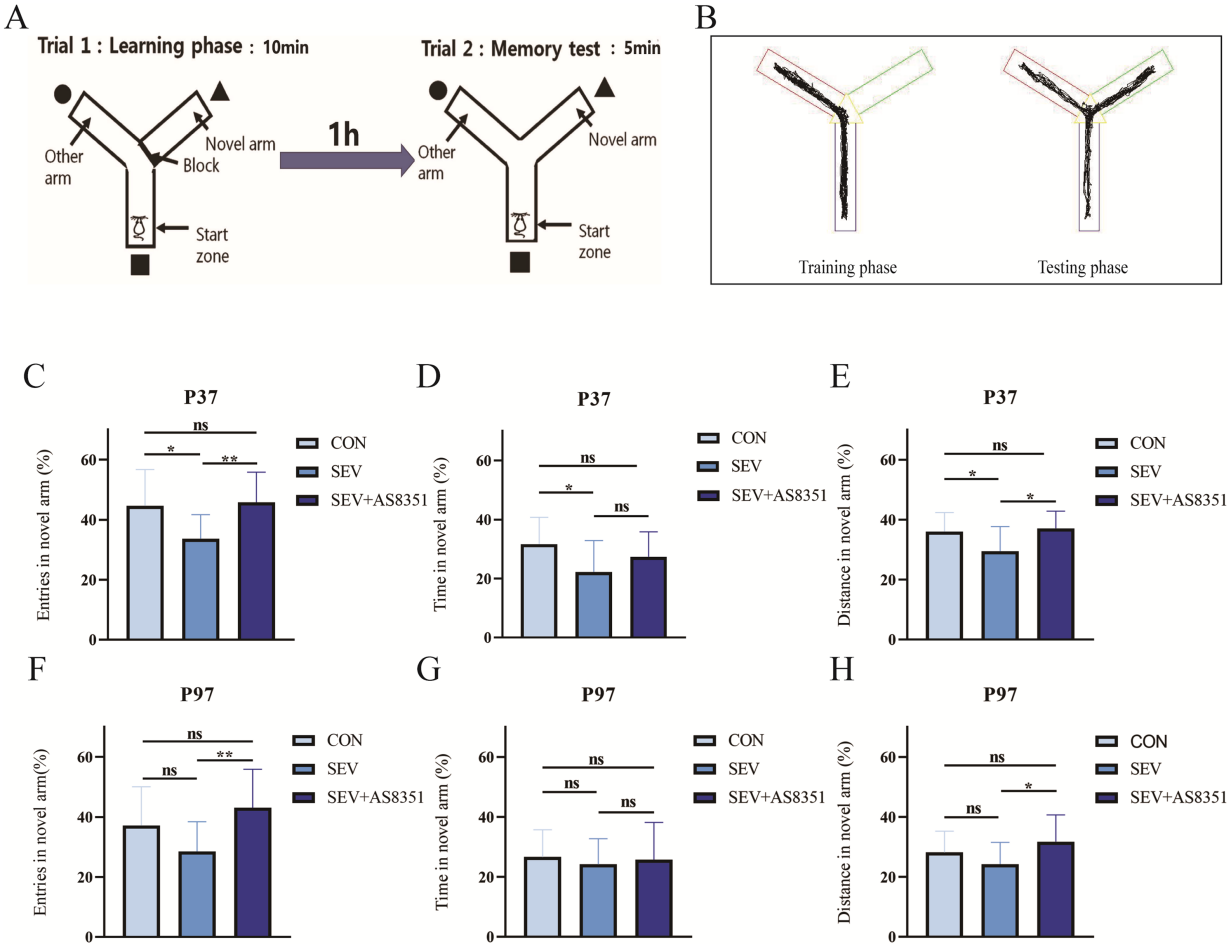
AS8351 partially restored the memory impairment in neonatal rats exposed to sevoflurane in Y maze. **(A)** Schematic diagram of Y maze experiment; **(B)** track diagram of training stage and Track diagram of test stage; **(C)** at P37, the percentage of entries in novel arm of the rats in the Y maze experiment (%); **(D)** at P37, the percentage of exploration time in novel arm of the rats in the Y maze experiment (%); **(E)** at P37, the percentage of exploration distance in novel arm in the Y maze experiment; **(F)** at P97, the percentage of entries in novel arm of the rats in the Y maze experiment (%); **(G)** at P97, the percentage of exploration time in novel arm of the rats in the Y maze experiment (%); **(H)** at P97, the percentage of exploration distance in novel arm in the Y maze experiment; Some rats were died during the early modeling and intraperitoneal injection. Data of over-active and under-active individuals were excluded during the experiment, finally, CON group (*n* = 13), SEV group (*n* = 14), SEV + AS8351 group (*n* = 14) at P37 and CON group (*n* = 17), SEV group (*n* = 16), SEV + AS8351 group (*n* = 16) at P97. *p < 0.05, **p < 0.01, ***p < 0.001.

The capacity of memory and learning was assessed using the Y maze. The experimental operation is shown in Figure 1A.

Y mazes can be used to evaluate the short-term memory of rodents and this experiment is a measure of working memory in rats. The Y maze consists of three arms spaced at a fixed angle of 120 and connected by a triangular area, each arm, s length is 42.5 cm, width is 14.5 cm and height is 22.5 cm. The experiment was divided into two phases: the training phase and the test phase. During the training phase, we use an opaque baffle to close one arm as a novel arm. The rats were allowed to enter the maze from one of the remaining two arms (as the initial arm) and move freely in the maze for 10 min, then put the rats back into the cage and wipe the maze with 75% ethanol solution when the training phase is finished. 1 h later, we start the test phase: the novel arm was opened and the rats were allowed to enter the maze from the end of the initial arm and move freely in the maze for 5 min. The smart 3.0 Behavioral Recording Analysis system (Panlab company, Spain) was used to record and calculate the percentage of entris, percentage of time, and percentage of distance explored by rats in novel arm.

### 2.4 Morris water maze (MWM) experiment

**Figure 2:**
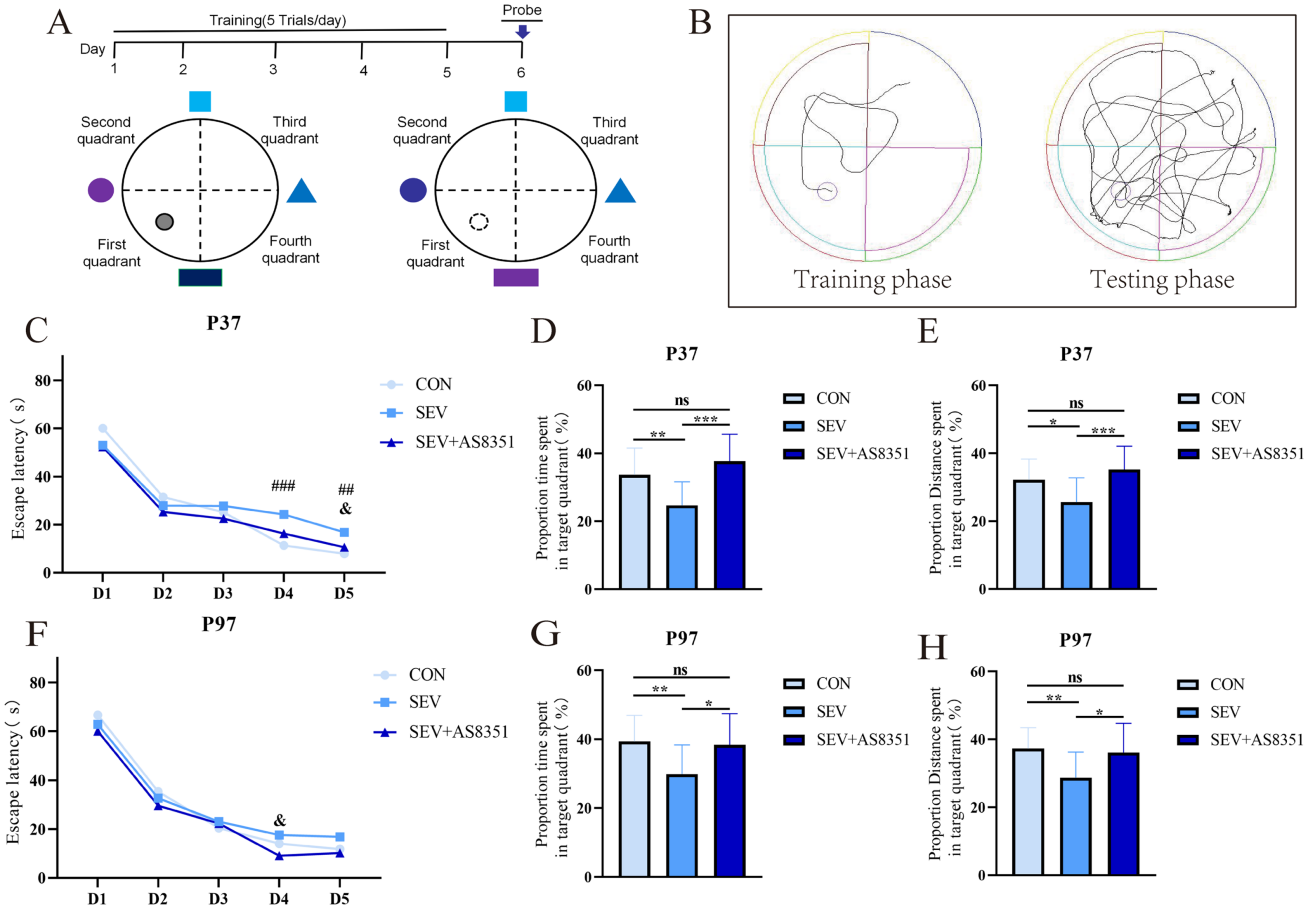
AS8351 partially restored the memory impairment in neonatal rats exposed to sevoflurane in MWM. **(A)** Schematic diagram of MWM; **(B)** track diagram of MWM experiment training stage and track diagram of MWM experiment stage; **(C)** the escape latency from day 1 to day 5 of MWM at P37; **(D)** the proportion time spent in target quadrant (%) in 90s after entering the water on the 6th day of MWM at P37; **(E)** the proportion distance spent in target quadrant (%) in 90s after entering the water on the 6th day of MWM at P37; **(F)** the escape latency from day 1 to day 5 of MWM at P97; **(G)** the proportion time spent in target quadrant (%) in 90s after entering the water on the 6th day of MWM at P97; **(H)** the proportion distance spent in target quadrant (%) in 90s after entering the water on the 6th day of MWM at P97; Some rats were died during the experiment, the overactive and underactive rats were excluded during the experiment, the remaining rats were included to analyze: At P37, CON group (*n* = 16), SEV group (*n* = 17), and SEV + AS8351 group (*n* = 15); CON group (*n* = 13), SEV group (*n* = 19), and SEV + AS8351 group (*n* = 15). Intra-group comparison of MWM training stage: *### p* < 0.001, *## p* < 0.01 SEV group and CON group; *& p* < 0.05 SEV group and SEV + AS8351 group. MWM test stage: *p < 0.05, **p < 0.01, ***p < 0.001.

Morris Water Maze Test was performed to evaluate the spatial memory and learning abilities of rats in the present study. The experimental operation diagram is shown in Figure 2A.

Rodents have a natural aversion to water, and MWM is a behavioral experiment that uses this characteristic to force them to swim and find hidden underwater platforms through learning and memory. The circular tank was 120 cm in diameter and 60 cm in height, filled with warm water at 23 ± 2°C to the depth of 30 cm. The maze was divided into four equal quadrants (First quadrant, Second quadrant, Third quadrant and Fourth quadrant). The escape platform with a diameter of 15 cm was situated the middle of the First quadrant and 2 cm beneath the surface of opaque water. A tracking system was used to record the movement of animals in the pool and light was blocked to avoid interference from the light source on animals and image acquisition. Day 1 ~ Day 5 was a positioning navigation experiment. The rats faced the maze wall at the midpoint of the four quadrants when entering the water, and the maximum time for the rats to find and climb the platform was set to be 90 s. The exact time the rats climbed onto the platform were recorded as the escape incubation period, and the rats were allowed to rest on the platform for 10 s, and if them did not reach the platform within 90 s, them were led to the platform and rested for 10 s. Day 6 was a space exploration experiment. At this time, the platform in the water is removed, and the rats were placed opposite the quadrant where the original platform was located and freely explored for 90 s. The number of times the rat platform has crossed, the exploration time in the quadrant where the platform is located, the percentage of time (%) and the percentage of distance (%) of the rats exploring in the quadrant where the platform was located during the period were calculated by behavioral recording system.

### 2.5 Western blot (WB)

The expression of BDNF and PSD95, histone methylation marker H3K4me3, and histone demethylase KDM5B in the hippocampal of each group of rats were detected by Western blot after MWM. Six rats in each group were randomly selected and intraperitoneally injected with 1% pentobarbital 50 mg/kg. The hippocampal tissue was isolated immediately after deep anesthesia was obtained. After weighing the hippocampus, the tissue was added to the lysate at the ratio of W:V = 1:5, mixed and lysed, centrifuged at 12000 rpm at 4°C for 15 min, and the supernatant was extracted. Protein quantification was performed using the Bicinonic acid (BCA). That is, Bovine serum albumin (BSA) standard is diluted with a gradient, the supernatant sample to be tested were added to a 96 well plate, BCA working solution added and reacted in the dark at 37°C for 30–60 min, the absorbance of the sample was measured at 562 nm wavelength by enzyme spectrometer, and the standard curve was established to determine the protein concentration of the sample. After mixing the sample volume: Loadingbuffer = 5:1, boil for 5 min, and then put on ice to cool. According to the experimental group, the hippocampus samples were gently added into the gel plate sample hole made of 10% separation glue solution and 5% concentrated glue and fixed in the electrophoresis tank. The electrophoresis buffer was poured into the electrophoresis apparatus and set to a stable state, and the pressure was adjusted to 80 V electrophoresis for 30 min. When the strip entered the concentrated glue, the pressure was adjusted to 120 V electrophoresis for 2 h. After gel electrophoresis, the protein bands separated from the gel are transferred to suitable cut Polyvinylidene fluoride (PVDF) membrane by transfer electrophoresis. After soaking in 100% methanol for 2–3 min, rinse with water and electric transfer solution for 2 min × 2 times and soak in the configured electric transfer solution. The excess gel plate was removed according to the molecular weight of the target protein against Marker and made into a “sandwich,” then put it into the transfer electrophoresis tank containing transfer buffer. Connect the electrodes and set the current to 65 V, and the membranes were washed with Tris buffered saline Tween (TBST) after 2 h of transfer, then the sealing liquid was closed on a shaker at room temperature for 1 h. the membranes were incubated overnight at 4°C with the following primary antibodies: BDNF (28205-1-AP, Proteintech, China), PSD95 (20665-1-AP, Proteintech, China), H3K4me3 (ab213224, Abcam, USA), KDM5B (ab181089, Abcam, USA) and *β*-actin (Proteintech, China), dilution and concentration used were optimized according to the antibody protocol instructions. After washes, the membranes were incubated with horseradish peroxidase-conjugated anti-rabbit secondary antibody (Beyotime Biotechnology, China) for 1 h at room temperature. The Enhanced chemiluminescence (ECL) and ChemiDoc MP chemiluminescence imaging system (Bio-rad, United States) at room temperature showed protein bands. The ImageJ and SPSS 29.0 software were used for analysis.

### 2.6 Transmission electron microscope (TEM)

In order to observe the effect of sevoflurane exposure on the hippocamphal synaptic structure of rats in the neonatal period, five rats in each group were randomly selected to anesthesia after MWM. Then, the brain tissues of the hippocampus (1.0 mm3) were dissected out. Post-fix in 2.5% glutaraldehyde (Spi-Chem Inc., USA) at 4°C for 2–4 h, and then rinsed with 0.1 M phosphoric acid bleach solution for 15 min × 3 times. Fixed at 4°C for 2 h. The tissue blocks were gradient dehydrated with alcohol for 15 min each time, and then soaked with 100% propylene oxide. The tissue containing propylene oxide was mixed with the embedding solution at 2:1 and left for 2 h at 37°C. The propylene oxide and the embedding solution were mixed at a ratio of 1:2 and allowed to stand at 37°C for 3 h. The tissue blocks were impregnated with pure embedding solution and stand for 3–4 h at 37°C, overnight at room temperature. Then the tissue block buried in the burial plate, stand at 68–70°C for 48 h. After the resin was completely polymerized, the embedded block was rough repair, and 70 nm sections were cut by ultramicrotome (Leica, Vienna, Austria). Ultrathin sections were stained with 3% uranium acetate saturated with 2.7% lead citrate solution. The ultrastructure of hippocampal synapses were observed and photographed by transmission electron microscopy (HITACHI, Japan), the length of the postsynaptic membrane, the thickness of dense layer of the postsynaptic membrane, the width of the synaptic gap are measured by Image-pro plus 6.0 software.

### 2.7 Statistical methods

All data were expressed as mean ± SD. SPSS 29.0 statistical software and Graphpad Prism 8 software were used for all statistical analysis. Data among multiple groups were compared by one-way ANOVA, repeated measure ANOVA was used for the training phase of Morris water maze experiment, Significant Difference (LSD) method was used for pairwise comparison of data between groups. Differences were considered statistically significant if *p* < 0.05.

## 3 Results

### 3.1 Repeated exposure to sevoflurane in neonatal rats causes long-term memory impairment, and KDM5B inhibitors can improve the memory impairment caused by sevoflurane exposure

To verify whether exposure to sevoflurane for 4 h on P7, P14 and P21 caused long-term cognitive impairment, we conducted Y-maze experiment to test the long-term memory of rats. Our experiment found that at P37, the percentage of entries in novel arm (%), the percentage of exploration time in novel arm (%), and the percentage of exploration distance in novel arm (%) in SEV group were lower than CON group (*p* < 0.05), as shown in Figures 1C–F. Continuous treatment with KDM5B inhibitors for 15 days after repeated exposure to sevoflurane, the percentage of exploration time in novel arm (%) was increased to the SEV group (*p* < 0.01), and the percentage of exploration distance of the novel arm (%) also increased compared to the SEV group (*p* < 0.05), as shown in Figures 1C,E. We also found that at P97, there was no significant difference in the percentage of entries in novel arm (%), the percentage of exploration time in novel arm (%), and the percentage of exploration distance in novel arm (%) between the SEV group rats and the CON group rats, as shown in Figures 1F–H. However, compared with the rats in the SEV group, the percentage of entries in novel arm (%) of the SEV + AS8351 group rats were increased (*p < 0.01*), and the percentage of exploration distance in novel arm (%) also increased (*p < 0.05*), as shown in Figures 1F,H.

These results indicated that repeated exposure to sevoflurane in neonatal rats caused working memory impairment in infancy, but did not reduce working memory ability when they are adults. KDM5B inhibitors can improve the long-term working memory decline in infancy induced by repeated sevoflurane exposure in neonatal rats.

To further verify whether sevoflurane exposure causes long-term cognitive impairment in the developing brain, we also performed the Morris water Maze experiment on rats, which was performed the day after the Y maze experiment. We found that the Mauchly’s test of sphericity had statistical significance at P37 (*p* < 0.01), and the escape latency of rats in each group decreased day by day from day 1 to day 5 (days: *F* = 126.992, *p* < 0.001; groups×days: *F* = 2.075, *p* < 0.05). On day 4 of the training phase, the escape latency in SEV group was longer than CON group (*p* < 0.001). On day 5 of the training phase, the escape latency of the SEV group was longer than CON group (*p* < 0.01) and SEV + AS8351 group (*p* < 0.05), as shown in Figure 2C. The platform was removed on day 6, the proportion time spent in target quadrant (%) was significant differences (*F* = 6.540, *p* < 0.01) among all groups, and the proportion distance spent in target quadrant (%) was also significant differences (*F* = 4.414, *p* < 0.01). Compared with the SEV group, the CON group had a difference in the proportion time spent in target quadrant (%) (*p* < 0.01), and also had a difference with the SEV + AS8351 group (*p* < 0.001), as shown in Figure 2D. Compared with the SEV group, the proportion distance spent in target quadrant (%) of the CON group was different (*p* < 0.05), and the CON group was also different from the SEV + AS8351 group (*p* < 0.001), as shown in Figure 2E.

Our experiment also found that the Mauchly’s test of sphericity statistical significance at P97 (*p* < 0.001), and the escape latency of rats in each group decreased day by day from day 1 to 5 (days: *F* = 219.109, *p* < 0.001; groups*days: *F* = 1.891, *p* < 0.05). On day 4 of the training phase, the escape latency of the SEV group was significantly longer than the SEV + AS8351 group (*p* < 0.05), but there was no significant difference between the SEV group and the CON group, as shown in Figure 2F. On day 6, there was significant difference between the proportion time spent in target quadrant (%) (*F* = 4.376, *p* < 0.01) and the proportion distance spent in target quadrant (%) (*F* = 4.220, *p* < 0.01). Compared with the SEV group, there were differences in the proportion time spent in target quadrant (%) and the proportion distance spent in target quadrant (%) in the CON group (*p* < 0.01), and differences in the proportion time spent in target quadrant (%) and the proportion distance spent in target quadrant (%) in the SEV + AS8351 group (*p* < 0.05), as shown in Figures 2G,H.

These results suggest that repeated exposure to sevoflurane in neonatal period leads to reduced long-term spatial memory ability in rats, and KDM5B inhibitors can improve the memory impairment induced by sevoflurane exposure.

### 3.2 Neonatal sevoflurane exposure increased the level of histone demethylase KDM5B in hippocampus of rats, and decreased the expression of H3K4me3, BDNF and PSD95–KDM5B inhibitor could partially reverse this change

**Figure 3:**
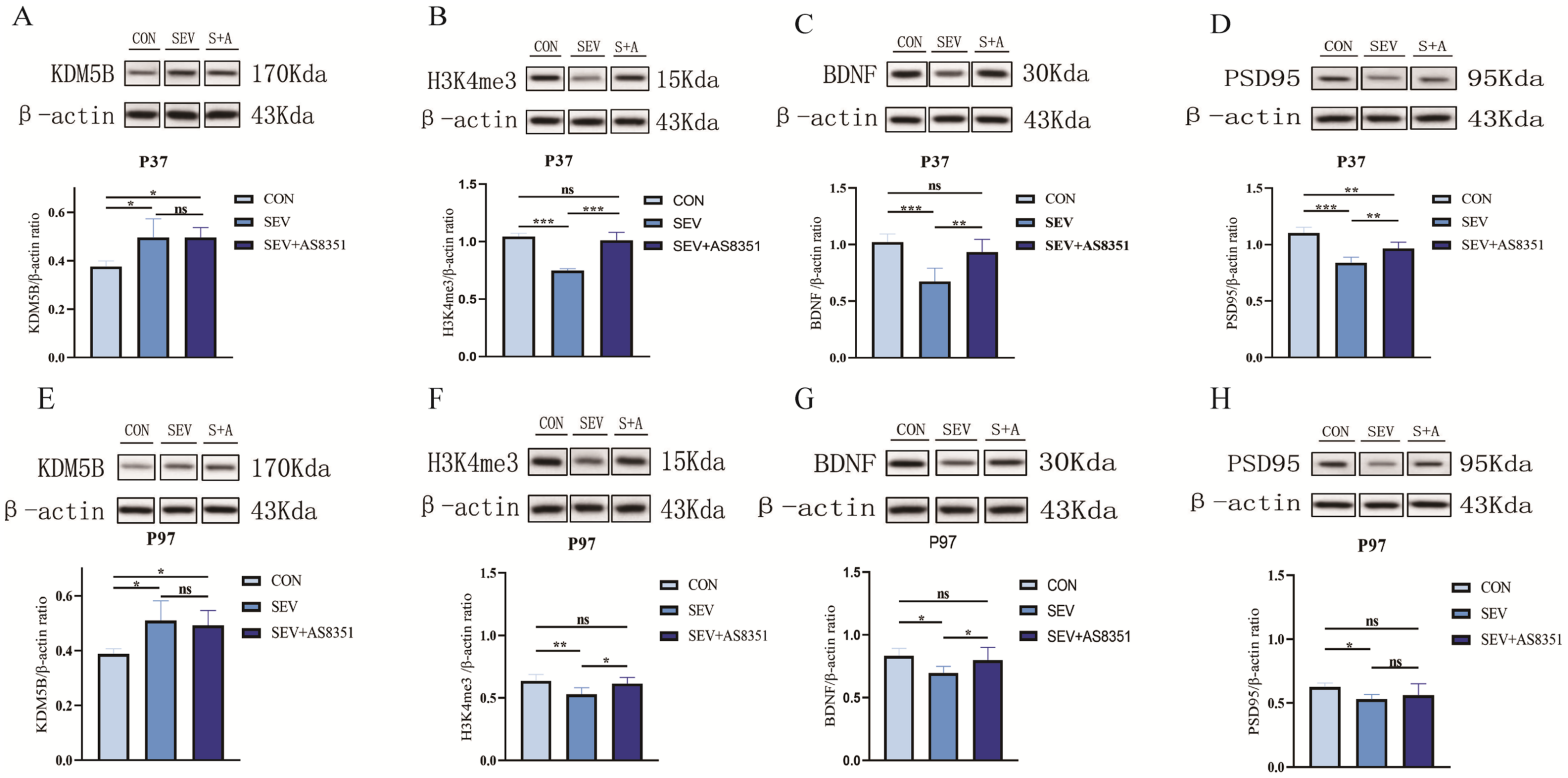
Neonatal sevoflurane exposure affected KDM5B expression in the hippocampus and leaded to changes in histone 3 lysine 4 trimethylated, downregulated the expression of synaptic associated proteins, and AS8351 partially restored the negative reaction caused by sevoflurane exposure. **(A)** Western blot analysis of KDM5B in the hippocampal of rats at P37; **(B)** western blot analysis of H3K4me3 in the hippocampal of rats at P37; **(C)** western blot analysis of BDNF in the hippocampal of rats at P37; **(D)** western blot analysis of PSD95 in the hippocampal of rats at P37; **(E)** western blot analysis of KDM5B in the hippocampal of rats at P97; **(F)** western blot analysis of H3K4me3 in the hippocampal of rats at P97; **(G)** western blot analysis of BDNF in the hippocampal of rats at P97; **(H)** western blot analysis of PSD95 in the hippocampal of rats at P97; (S + A is SEV + AS8351 group); (*n* = 6). *p < 0.05, **p < 0.01, ***p < 0.001.

In order to clarify the possible mechanism of long-term memory impairment caused by sevoflurane exposure in the neonatal period and the role of histone methylation modification in this process. After the behavioral experiment, we randomly executed six rats in each group and isolated their hippocampus, extracted total tissue protein, and detected the protein expression level in the hippocampus of the rats by WB experiment. We found that at P37, compared with the CON group, the expression of KDM5B in hippocampus of rats in SEV group and SEV + AS8351 group were significantly increased (*p* < 0.05), shown as Figure 3A. Compared with the SEV group, the expression of H3K4me3 in CON group and SEV + AS8351 group was significantly increased (*p* < 0.001), shown as Figure 3B. Compared with the SEV group, the expressions of BDNF, PSD95 in the hippocampus of CON group were significantly increased (*p* < 0.001), and the expressions of BDNF, PSD95 in SEV + AS8351 group were significantly increased (*p* < 0.01). Furthermore, the expression of PSD95 in SEV + AS8351 group was significantly increased compared with SEV group (*p* < 0.01), as shown in Figures 3C,D. These results indicated that repeated exposure to sevoflurane in neonatal rats can up-regulate KDM5B expression, down-regulate H3K4me3 expression, and down-regulate BDNF and PSD95 expression in juvenile rats. Posttreatment with KDM5B inhibitor had no significant effect on the expression level of KDM5B, but its use reversed the downregulation of H3K4me3 expression and synapse-associated protein expression induced by sevoflurane exposure.

We also found that at P97, compared with the CON group, the expression of KDM5B in hippocampus of SEV group and SEV + AS8351 group were significantly increased (*p* < 0.05), as shown in Figure 3E. Compared with the SEV group, H3K4me3 expression in CON group was significantly increased (*p* < 0.01), and SEV + AS8351 group was also significantly increased (*p* < 0.05), as shown in Figure 3F. Compared with the SEV group, the expressions of BDNF and PSD95 in CON group were significantly increased (*p* < 0.05), and the expressions of BDNF in SEV + AS8351 group were increased (*p* < 0.05), as shown in Figures 3G,H. These results indicated that repeated exposure to sevoflurane in neonatal rats can up-regulate KDM5B expression, down-regulate H3K4me3 expression, and down-regulate synapse-related protein expression in the hippocampus of adult rats. KDM5B inhibitors had no significant effect on the expression of KDM5B, but could partially reverse the negative reaction caused by sevoflurane exposure.

### 3.3 Sevoflurane exposure in neonatal period damaged the hippocampal synaptic structure, and KDM5B inhibitors had a certain protective effect on hippocampal synapses

**Figure 4:**
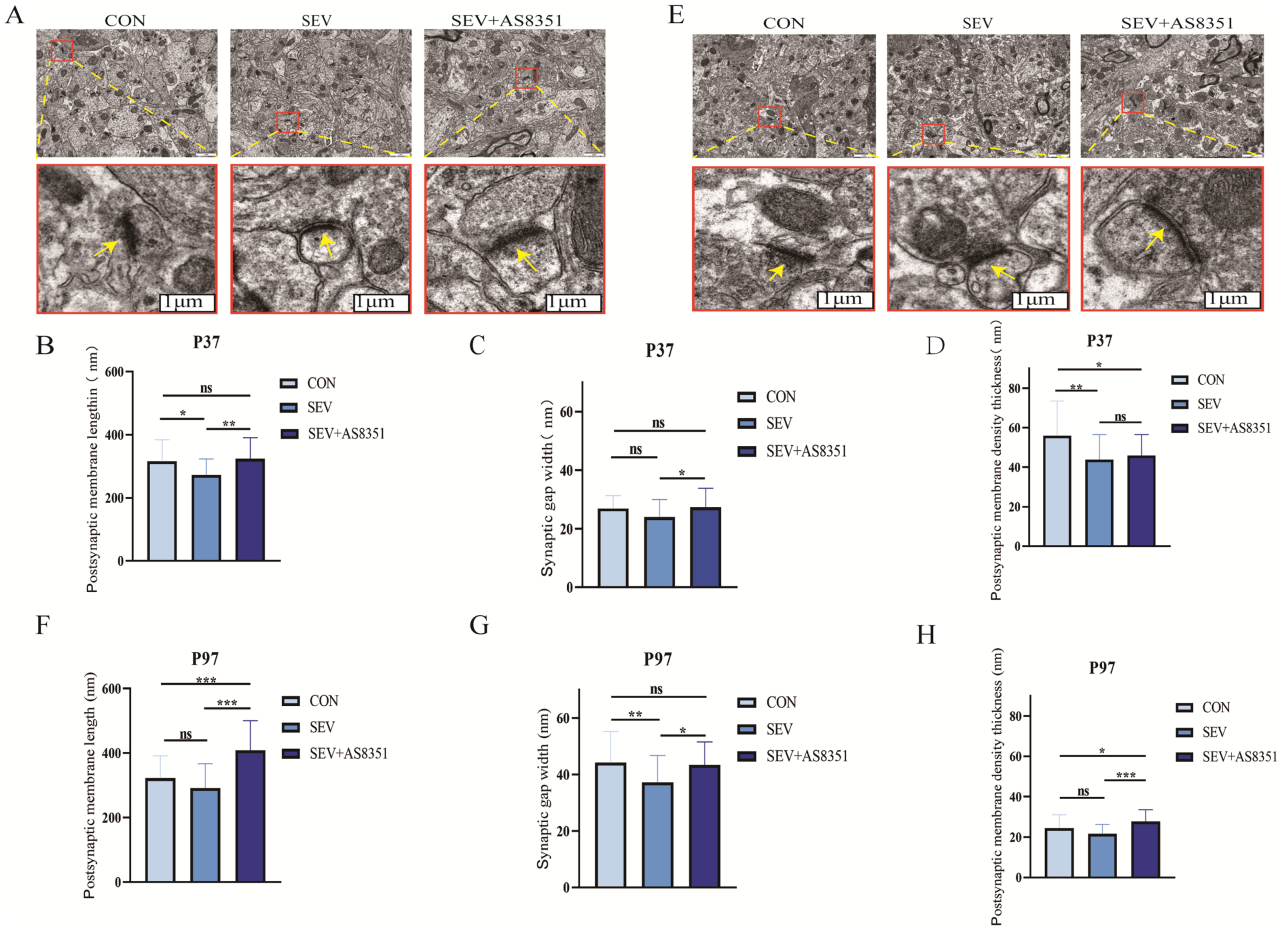
AS8351 protected the hippocampal synaptic structure of rats from neonatal sevoflurane exposure. **(A)** Representative TEM images of hippocampal synapses in each group at P37 (scale 1um); **(B)** statistical diagram of hippocampal postsynaptic membrane lengthin each group at P37; **(C)** statistical diagram of hippocampal synaptic gap width in each group at P37; **(D)** statistical diagram of postsynaptic membrane density thickness in each group at P37; **(E)** representative TEM images of hippocampal synapses in each group at P97 (scale 1um); **(F)** statistical diagram of hippocampal postsynaptic membrane lengthin each group at P97; **(G)** statistical diagram of hippocampal synaptic gap width in each group at P97; **(H)** statistical diagram of postsynaptic membrane density thickness in each group at P97; (*n* = 5). *p < 0.05, **p < 0.01, ***p < 0.001.

In order to further determine whether neonatal sevoflurane exposure caused damage to the hippocampal synaptic structure of rats, we decapitated the rats immediately after the behavioral experiment, isolated the hippocampus quickly and observed the ultrastructure and morphological changes of hippocampal synapses by transmission electron microscopy. We found that there were some differences in the ultrastructure of hippocampal synapses in different groups at P37 and P97. Compared with the SEV group, the synaptic structure and synaptic gap in the hippocampus of rats in the CON group were clearer, the postsynaptic membrane was significantly longer and the dense layer of the postsynaptic membrane was thicker. The synaptic structure of the SEV + AS8351 group was similar to that of the CON group, as shown in Figures 4A,E (yellow arrows point to synapses in the figure). At P37, compared to the CON group, the postsynaptic membrane (*p* < 0.05) and the dense layer of the postsynaptic membrane (*p* < 0.01) were significantly decreased in SEV group, the dense layer of the postsynaptic membrane (*p* < 0.05) was significantly decreased in SEV + AS8351 group. Compared to the SEV group, the postsynaptic membrane (*p* < 0.01) and the synaptic gap (*p* < 0.05) were significantly increased in SEV + AS8351 group, as shown in Figures 4B–D. At P97, compared to the CON group, the synaptic gap was significantly decreased (*p* < 0.01), postsynaptic membrane and postsynaptic membrane density had no significant difference in SEV group, the postsynaptic membrane (*p* < 0.001) and the dense layer of the postsynaptic membrane (*p* < 0.05) were significantly increased in SEV + AS8351 group. Compared to the SEV group, the postsynaptic membrane (*p* < 0.001), the synaptic gap (*p* < 0.05) and the dense layer of the postsynaptic membrane (*p* < 0.001) were significantly increased in SEV + AS8351 group, as shown in Figures 4F–H. These results indicate that repeated exposure to sevoflurane in neonatal period leads to the destruction of hippocampal synaptic ultrastructure, and KDM5B inhibitors can reduce the negative synaptic ultrastructure changes induced by sevoflurane exposure.

## 4 Discussion

Rats have a 24-h circadian rhythm that is most active at night, but they also move and eat during the day. In addition, they are more active in the morning than in the afternoon. Changes in circadian rhythms can affect measures of behavior. Sleep deprivation leads to cognitive impairment and circadian dysfunction, and the hippocampus is involved in this process (Giri et al., 2024; Hu et al., 2024). To rule out the negative effects of circadian rhythm changes on this study, we modulated the circadian rhythms in rats: For 12 h of light (20:00 -- 8:00) and 12 h of darkness (8:00 -- 20:00), and rats were exposed to sevoflurane from 8:00 a.m. to 12:00 a.m.

### 4.1 Neonatal anesthesia exposure impaired long-term synaptic plasticity and memory

The experiments showed that repeated exposure to sevoflurane in the neonatal period of P37 decreased the expression of BDNF and PSD95 in the hippocampus of rats, hippocampal neurons showed negative structural changes such as narrow width of postsynaptic membrane and thin dense layer of postsynaptic membrane. Then the behavioral experiments such as Y Maze and Morris Water maze showed that the learning and memory ability of rats decreased in juvenile. Synaptic plasticity is considered to be an important part of learning and memory (Magee and Grienberger, 2020; Goto et al., 2021; Kozachkov et al., 2022), which is mainly divided into functional plasticity and structural plasticity. Brain-derived neurotrophic factor (BDNF) is a kind of neurotransmitter present in nearly all Brain regions (Devlin et al., 2021). It primarily acts in the presynaptic and postsynaptic regions and plays a crucial role in regulating the survival and growth of neurons, as well as synaptic efficiency and plasticity, and is heavily involved in the formation and maintenance of memory by promoting synaptic consolidation (Camuso et al., 2022). Postsynaptic density protein 95 (PSD95) is the most abundant scaffold protein in excitatory postsynaptic density. Postsynaptic density protein95 (PSD95) interacts with a variety of key molecules in neuronal function and exerts an effect on the organization of postsynaptic membrane, thereby limiting synaptic plasticity and stabilizing neural circuits, ultimately involved in basic neural processes such as neuronal plasticity and memory (Bustos et al., 2017). Changes in the expression of BDNF and PSD95 can affect the function and structure of synapses, and thus have an important impact on learning and memory. Dong et al. showed that repeated inhalation of Sevoflurane increased the expression of brain-derived neurotrophic factor precursor (proBDNF), while the down-regulations of synapse-related proteins such as BDNF, tissue plasminogen activator (tPA) and phosphorylated tyrosine kinase receptor B (p-TrkB), resulting in a decrease in the density of hippocampal dendritic spines, and eventually, learning and memory dysfunction occurs (Dong et al., 2020). In addition, Li et al. also found that early exposure to sevoflurane in newborn rats significantly reduced the expression of PSD95 in the developing hippocampus, reduced the density of hippocampal synapses, and impaired hippocampal dependent fear memory (Li et al., 2022). This is consistent with our experimental results, suggesting that BDNF and PSD95 signaling are closely related to the molecular mechanism of long-term memory impairment induced by early sevoflurane exposure in rats.

Our experiments found that neonatal sevoflurane exposure downregulated the expression of PSD95 in hippocampus at P97, but TEM showed that the structure of the postsynaptic membrane was restored, and cognitive function was recovered in the Y-maze test, it is inconsistent with P37. Someone found that newborn mice repeated inhalation of isoflurane for 24 h, hippocampal BDNF and synapse (Syn) were reduced significantly, hippocampal neuron apoptosis and damage. The negative changes can be partially recovered exceed P35, but they still show spatial cognitive impairment. At P65, almost all the adverse changes caused by isoflurane inhalation were recovered, and the behavioral performance was not different from ordinary mice (Liu et al., 2017). This is consistent with our results. This performance may be due to acute nerve cell damage and decrease the learning and memory ability caused by exposure to sevoflurane in the developing brain, but some function and structure of nerve cells were recovered or partially recovered during development, Therefore, the learning and memory abilities of neonatal rats exposed to sevoflurane are no different from those of normal rats in adulthood. As is well known, The postsynaptic dense layer is a part of the postsynaptic membrane, which is a dynamic cell compartment located the bottom of postsynaptic membrane (Okabe et al., 1999). A lot of proteins, such as PSD95, protein kinase C (PKC), calmodulin (CaM), a series of protein kinases the mitogen-activated protein kinase pathway (MAP kinase pathway), cAMP-dependent protein kinase, nitric oxide synthase (nNOS), working together to maintain the normal function and morphology of postsynaptic dense layer (Shen et al., 2023). Neonatal rat exposure to sevoflurane down-regulated PSD95 expression until P97, However, during the development, other proteins restored the morphology of the postsynaptic dense layer, the structure of the postsynaptic membrane was also restored, and their learning and memory abilities were similar to normal rats.

At P97, the results of Y maze experiment are not consistent with MWM experiment. The inconsistency of the detection principle and purpose of the two experiments may be the reason for the inconsistency of the results. Y maze novel arm exploration experiment was designed based on the natural habit of mice to actively explore new and different environments, and was mainly used to test the short-term spatial recognition memory ability of rodents. MWM experiment is an experiment which rodents are forced to swim to learn to find a platform hidden in water by using their water-hating nature. It is mainly used to test the long-term learning and memory ability of experimental animals in spatial positioning and object recognition (Ghafarimoghadam et al., 2022). Our experimental results showed that P37 rats showed both short-term memory and long-term memory damage, and P97 rats only had an effect on long-term memory, suggesting that sevoflurane exposure had a longer effect on long-term memory than short-term memory.

### 4.2 Repeated inhalation of sevoflurane in neonatal period down-regulated H3K4me3 in hippocampus

In the process of cell development, histone methylation not only affects gene transcription and new protein synthesis, but also causes cognitive function changes by altering synaptic plasticity. A study found that H3K4me3 was increased at the Bdnf promoters in aged rat, and showed aversive memory deficits (de Meireles et al., 2019). Other study showed that long-term ethanol treatment resulted in increased trimethylation of H3K4 and up-regulated PSD95 expression in rat cerebellum (Dulman et al., 2021). Those proved that BDNF and PSD95 were regulated by H3K4me3 and has an effect on memory. It was found that early exposure to midazolam, an intravenous anesthetic, persistently altered the expression of hippocampal chromatin and neural stem cell-related genes in mice, leading to reduced neurogenesis associated with impaired hippocampal dependent memory function (Doi et al., 2021). Rump et al. further found that midazolam exposure can change the H3K4 methylation level (Rump et al., 2022), and the reduction of H3K4 trimethylation level can cause abnormal growth and development of dendrites and affect memory formation (Timmerman et al., 2013). Therefore, we concluded that the change of H3K4 methylation level during midazolam anesthesia can cause memory damage. At present, it has not been reported in the literature whether sevoflurane exposure in the developing brain leads to long-term cognitive impairment by affecting the expression of H3K4me3 in the hippocampus, but our experimental results show that sevoflurane exposure in neonatal rats leads to the downregulation of H3K4me3 expression in the hippocampus, the destruction of synaptic structure, and the impairment of learning and memory ability during juvenile and adulthood. This suggests that sevoflurane has a similar effect on histone methylation in the hippocampus as midazolam.

### 4.3 Changes in the expression of histone demethylase KDM5B play a role in the long-term cognitive impairment induced by neonatal sevoflurane exposure

Studies have found that sevoflurane exposure during pregnancy up-regulates histone deacetylase 2 (HDAC2) and histone deacetylase 3 (HDAC3) levels in the hippocampus of the offspring, causing impairment of spatial learning and memory (Yu et al., 2020). Other studies have shown that HDAC2, HDAC4, HDAC7, HDAC8 and HDAC10 are widely up-regulated in the hippocampus of postoperative cognitive dysfunction (POCD) model of mice established by isoflurane (Yang et al., 2020). These studies suggest that inhalation narcotic exposure causes cognitive impairment by upregulating histone deacetylase levels. However, it has not been clear whether inhalation anesthetics affect cognitive function by affecting histone demethylase expression levels. Our study found that neonatal sevoflurane exposure up-regulated the expression of KDM5B in juvenile and adult rats. KDM5B is H3K4me2/3 specific demethylase. Our experimental results also found that the expression of H3K4me3 in the hippocampus of rats was downregulated and behavioral changes were observed, suggesting that the changes in histone demethylase induced by sevoflurane exposure in neonatal rats may be the cause of long-term cognitive impairment.

To further clarify elucidate the mechanism of these results, we observed the effects by post-treatment with histone demethylase inhibitors. The results showed that KDM5B inhibitors did not change the expression level of KDM5B after exposure to sevoflurane in neonatal rats, this is because KDM5B inhibitors can only inhibit the activity of the enzyme and have no effect on its expression, but can increase H3K4me3 (Chen X. et al., 2022). Satoru Matsuda et al. found that T-448, an inhibitor of the H3K4 demethylase LSD1, selectively inhibits LSD1 enzyme activity in primary rat embryonic neurons, increases H3K4 methylation levels and induces gene transcription in neurons. At the same time, they found that in mouse models of impaired function of the N-methyl-D-aspartate receptor (NMDAR), T-448 can increase H3K4 methylation in the brain and normalize expression levels of genes associated with neuroplasticity, thereby partially restoring the learning dysfunction of this mouse model (Matsuda et al., 2019). Recent studies have shown that upregulation of H3K4 methylation by oral KDM1A-specific inhibitors can rescues the defects of adult neurogenesis in the mouse models of Kabuki syndrome (KS), significantly improve the visuospatial learning and memory defect (Zhang et al., 2021). Consistent with there studies, we found that the reduction in H3K4 trimethylation, neuronal injury, and memory impairment induced by neonatal sevoflurane exposure can rescued by treatment with KDM5B inhibitor. These results suggest that regulate the function of histone demethylase can improve the cognitive deficits caused by abnormal gene transcription.

In our study, we observed a sustained inhibitory effect of AS8351 on KDM5B activity up to P97, 60 days after the final injection at P36. This prolonged effect may be related to the relatively stable nature of histone methylation modifications, specifically H3K4me3, which are known to have a relatively long half-life. Studies suggest that certain histone modifications, including H3K4me3, can persist over extended periods, contributing to long-lasting changes in gene expression and cellular memory. For example, research have reported that H3K4me3 can maintain its methylation status even in the absence of continuous enzymatic activity (Tran et al., 2022), potentially explaining the lasting cognitive effects observed in our model. We hypothesize that AS8351’s inhibition of KDM5B at early stages led to sustained changes in H3K4me3 levels, which could influence gene expression and contribute to enduring functional outcomes in hippocampal neurons. This hypothesis aligns with literature indicating that histone methylation changes can be stable over time and may not require constant inhibition to exert long-term effects.

## Data Availability

The original contributions presented in the study are included in the article/supplementary material, further inquiries can be directed to the corresponding author.
